# Supplementary material for: Zero Indirect Band Gap and Flat Bands in a Niobium Oxyiodide Cluster Material
Source: J Am Chem Soc. 2026 Jul 16;148(29):30861–72. doi: 10.1021/jacs.6c03891 (PMC13426306; doi:10.1021/jacs.6c03891)
Supplement: Supplementary file 1 [file ja6c03891_si_001.pdf]

Zero Indirect Band Gap and Flat Bands in a Niobium Oxyiodide Cluster Material – Supporting Information

Jan Beitlberger,[a] Mario Martin,[b] Marcus Scheele,[b] Marek Matas,[c] Carl P. Romao,*[c] Markus Ströbele,[a] and H.-Jürgen Meyer*[a]

[a] Section for Solid State and Theoretical Inorganic Chemistry, Institute of Inorganic Chemistry, Auf der Morgenstelle 18, 72076 Tübingen, Germany.

[b] Institute for Physical and Theoretical Chemistry, Eberhard-Karls-Universität Tübingen, Auf der Morgenstelle 18, 72076 Tübingen, Germany.

[c] Faculty of Nuclear Sciences and Physical Engineering, Czech Technical University in Prague, Czech Republic.

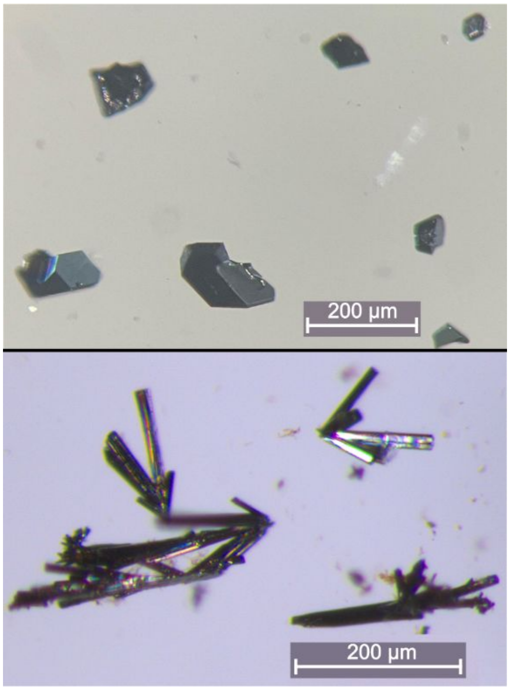

Fig. S1: Examples of block-shaped $Nb_6O_3I_{15}$ (top) and columnar $Nb_{11}O_6I_{24}$ (bottom) crystals

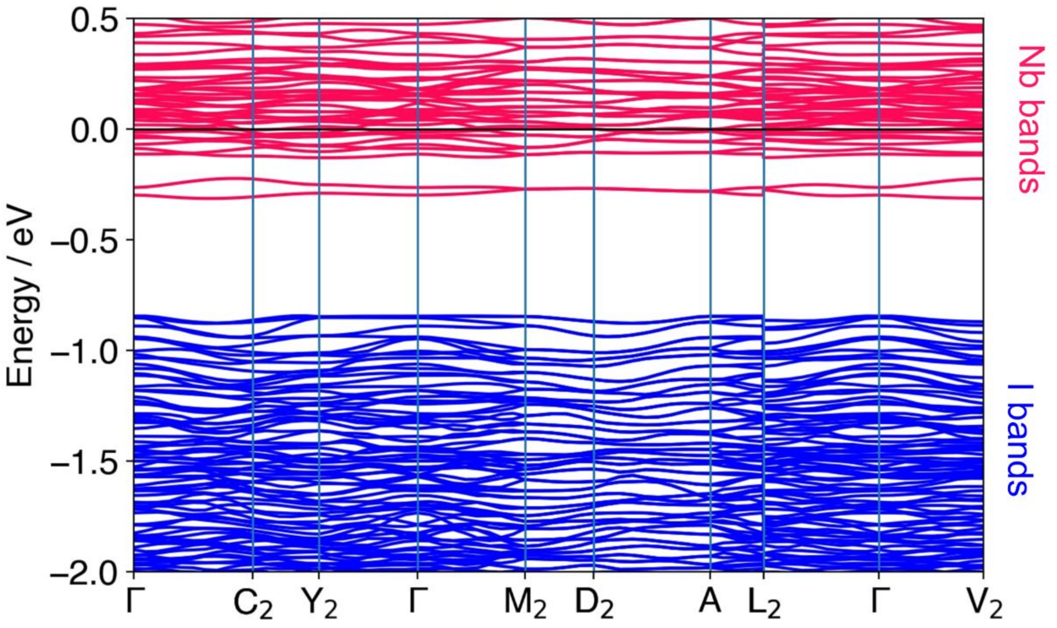

Fig. S2: Electronic band structure of $Nb_6O_3I_{15}$, with bands colored according to their main Nb or I character.

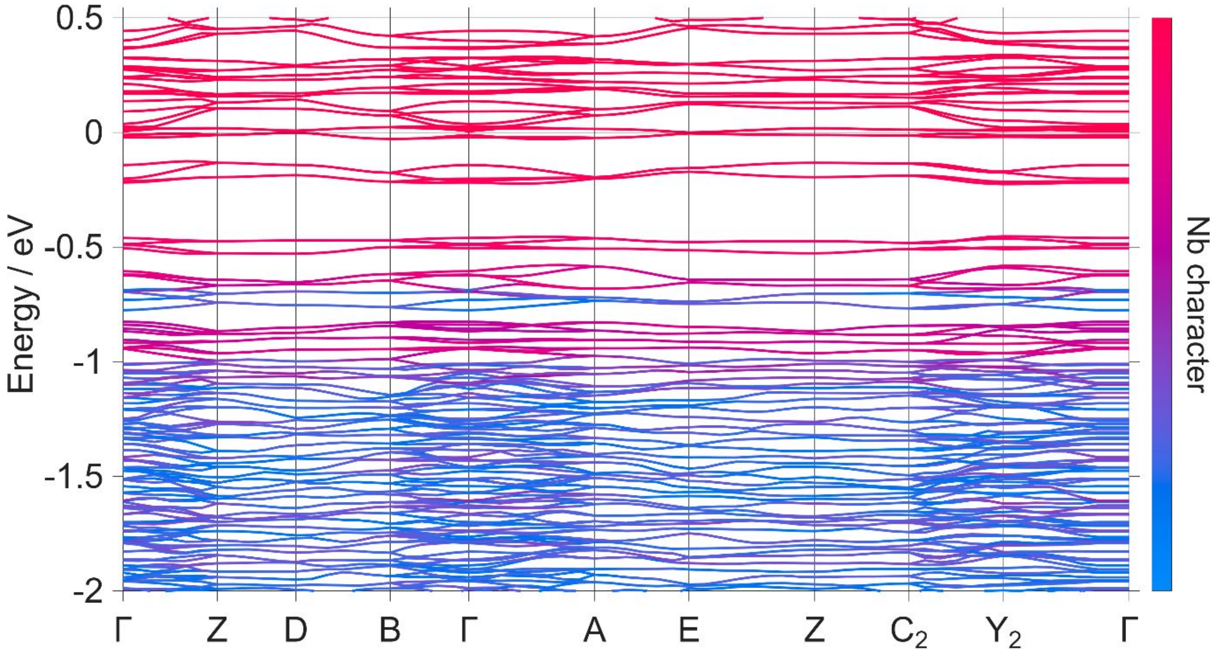

Fig. S3: Electronic band structure of $Nb_{11}O_6I_{24}$, with bands colored according to their Nb character.

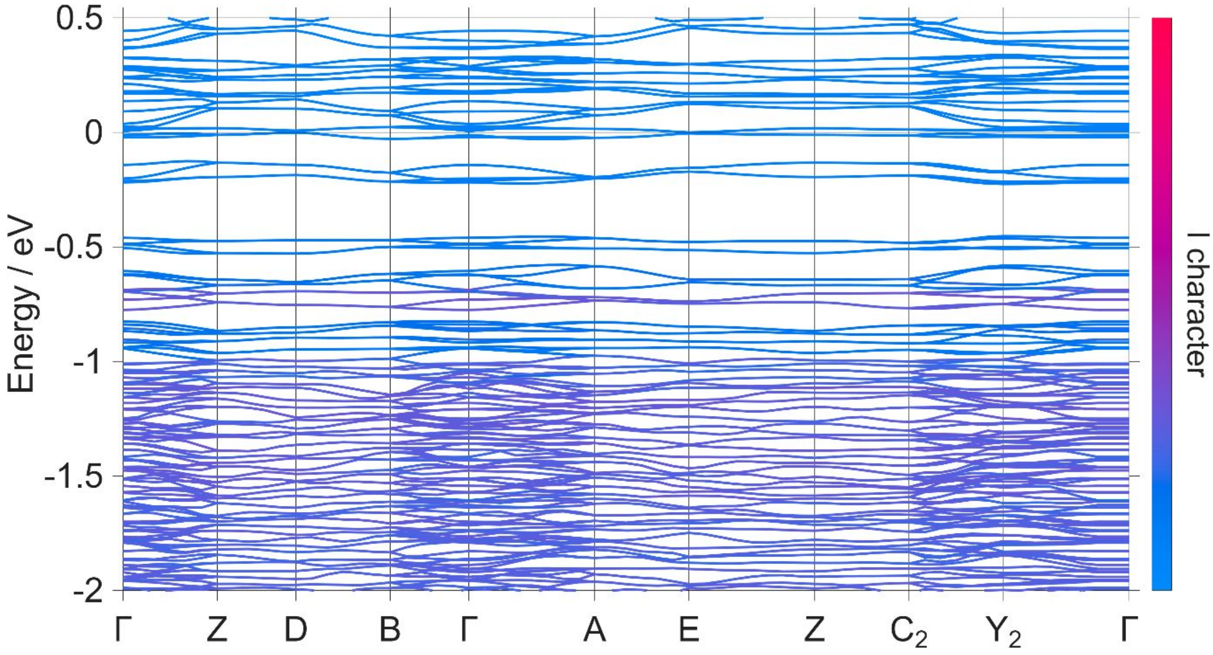

Fig. S4: Electronic band structure of $Nb_{11}O_6I_{24}$, with bands colored according to their I character.

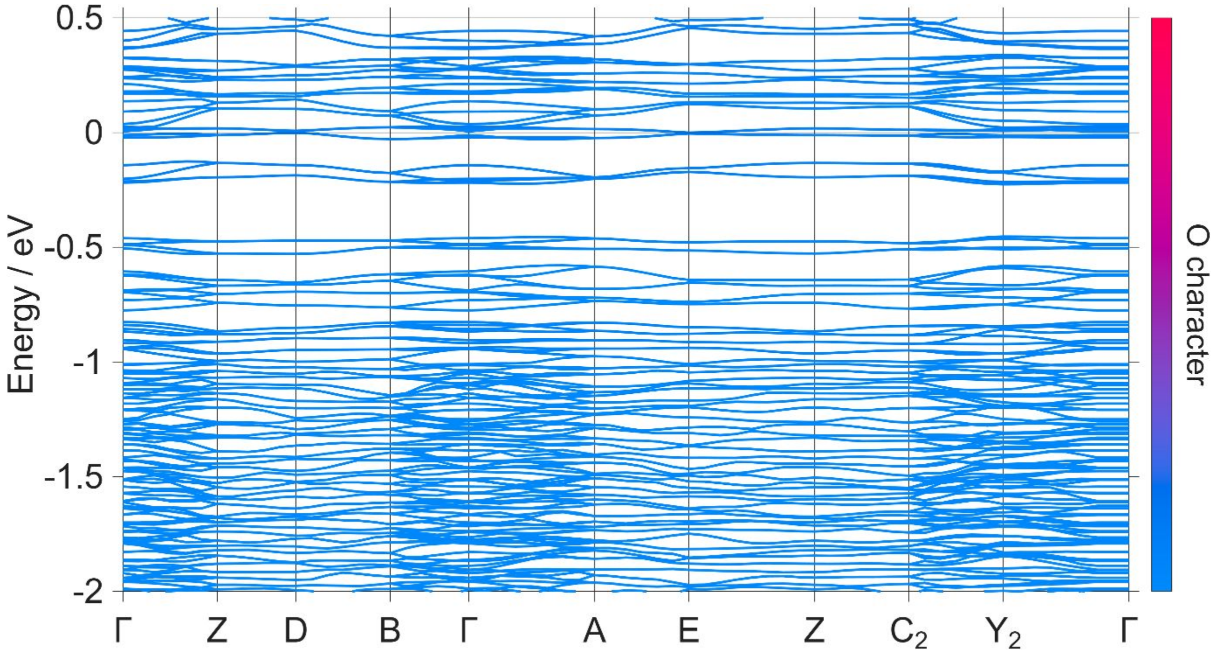

Fig. S5: Electronic band structure of $Nb_{11}O_6I_{24}$, with bands colored according to their O character.

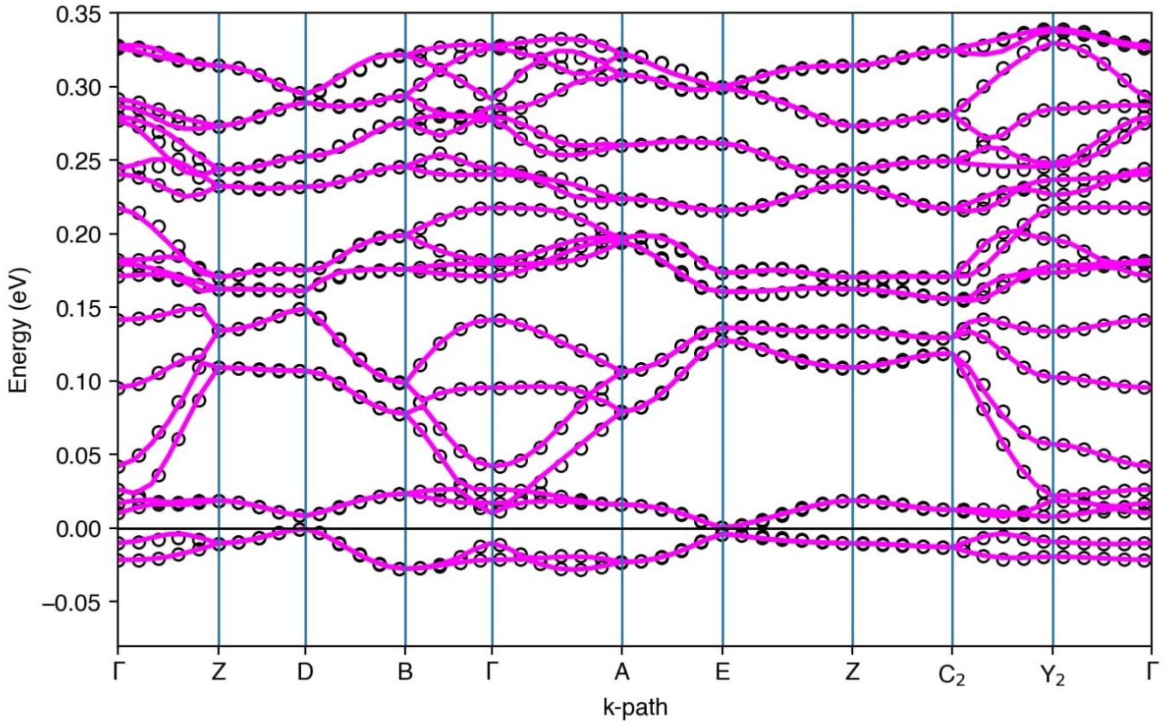

Fig. S6: Electronic band structure of $Nb_{11}O_6I_{24}$, with magenta lines showing the DFT-calculated bands and open circles showing the bands from the tight-binding model corresponding to the Wannier functions.

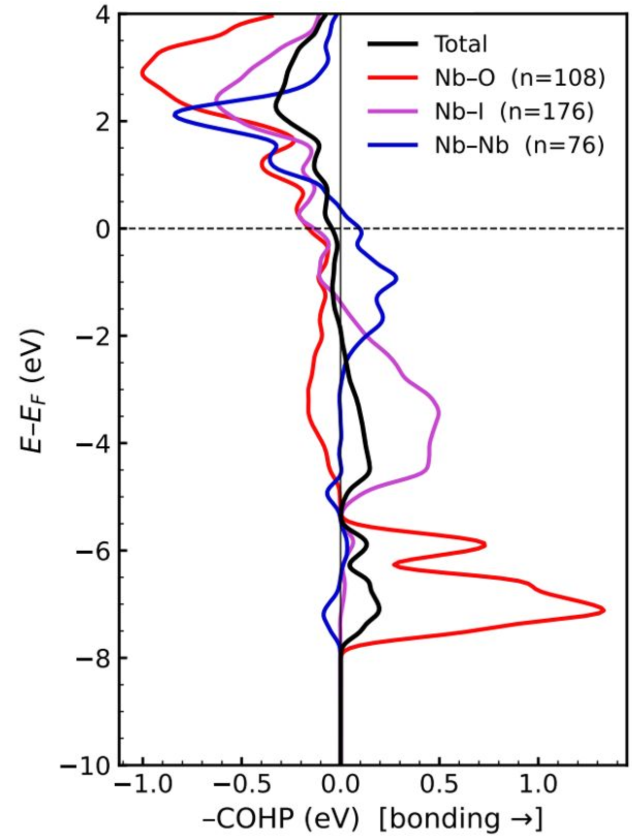

Fig. S7: Crystal orbital Hamilton populations (COHP) in $Nb_{11}O_6I_{24}$.

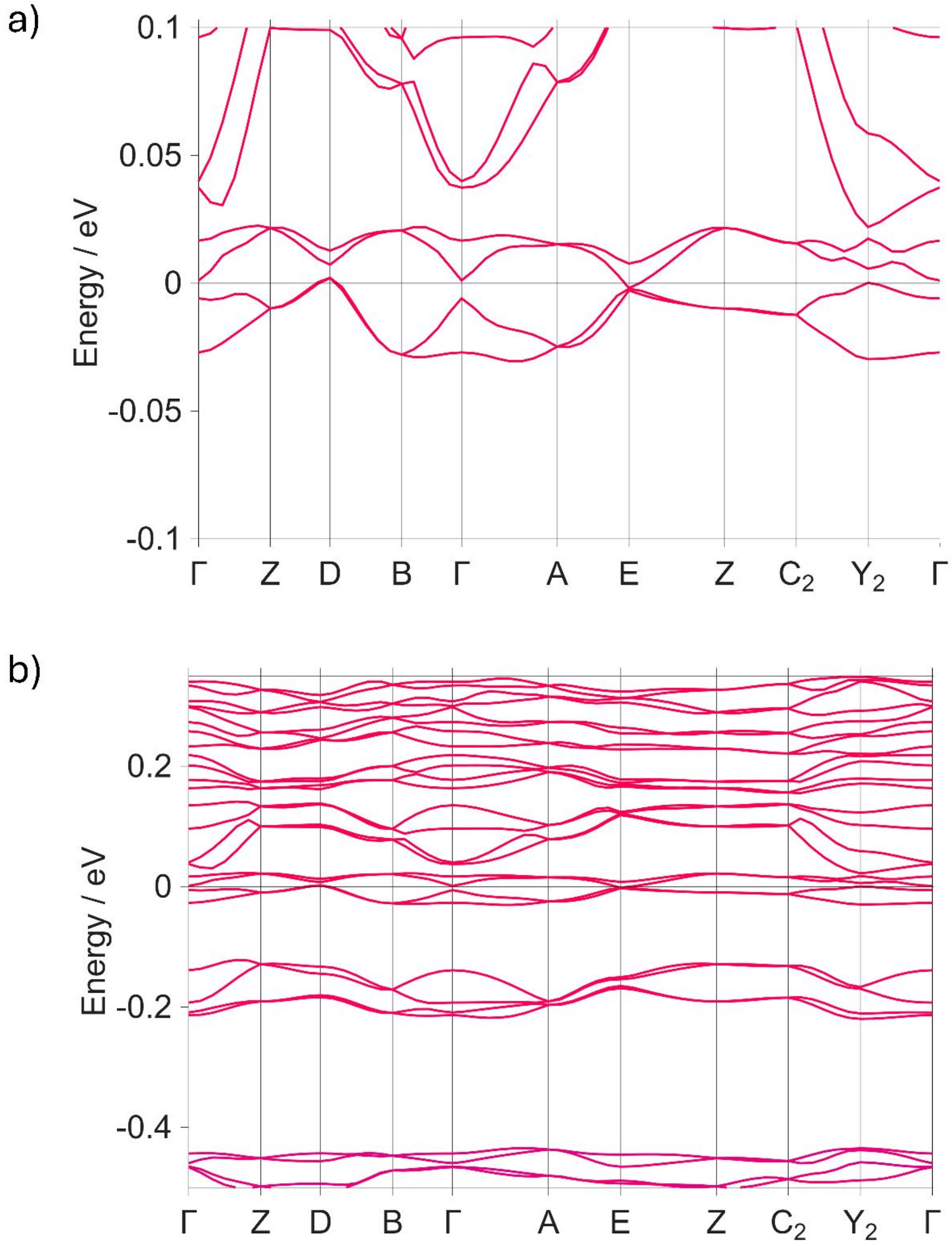

Fig. S8: Electronic band structure of $Nb_{11}O_6I_{24}$, calculated with spin–orbit coupling. A close-up of the bands near the Fermi energy is shown in (a), whereas a wider view is shown in (b).

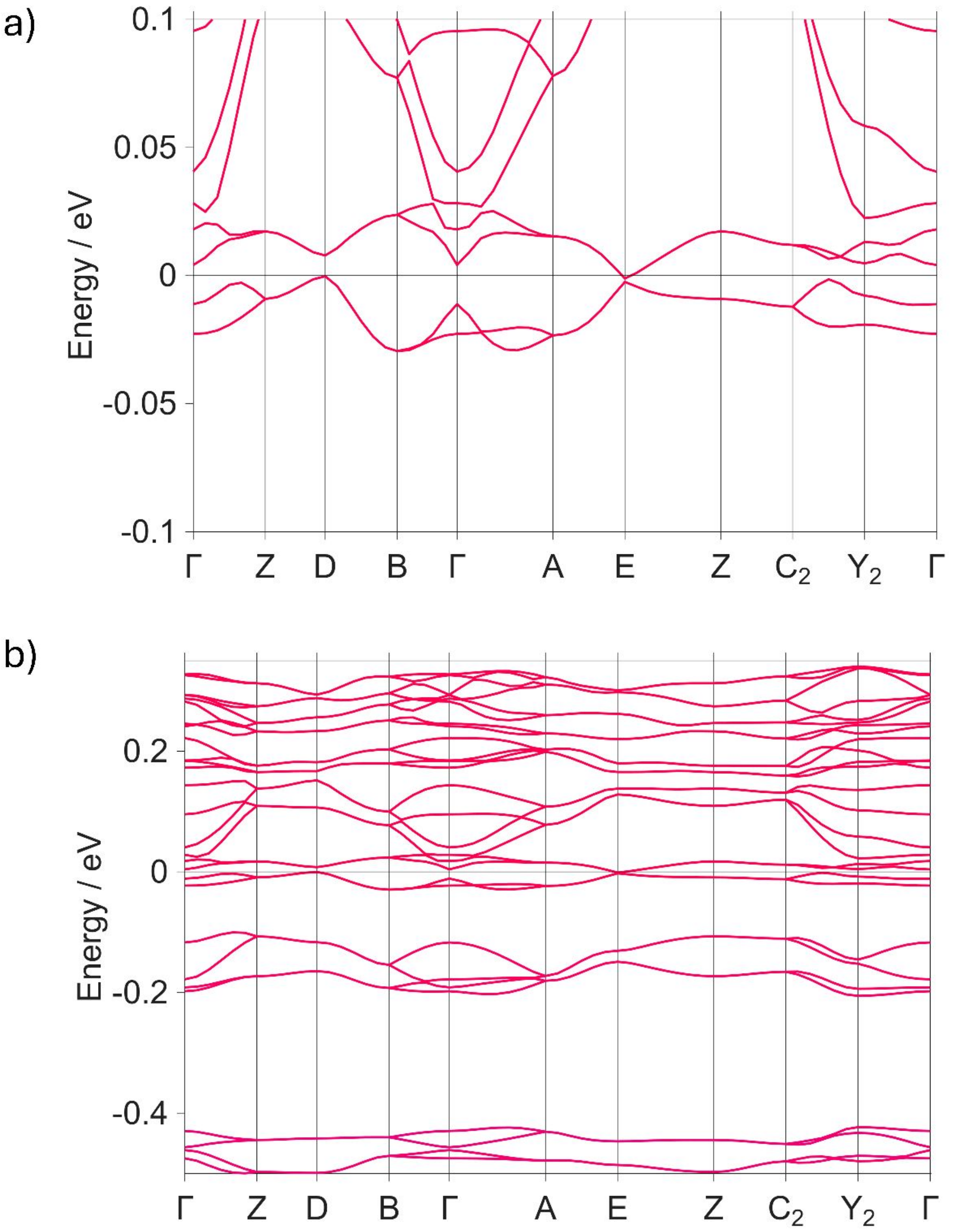

Fig. S9: Electronic band structure of $Nb_{11}O_6I_{24}$, calculated using the LDA exchange–correlation functional. A close-up of the bands near the Fermi energy is shown in (a), whereas a wider view is shown in (b).

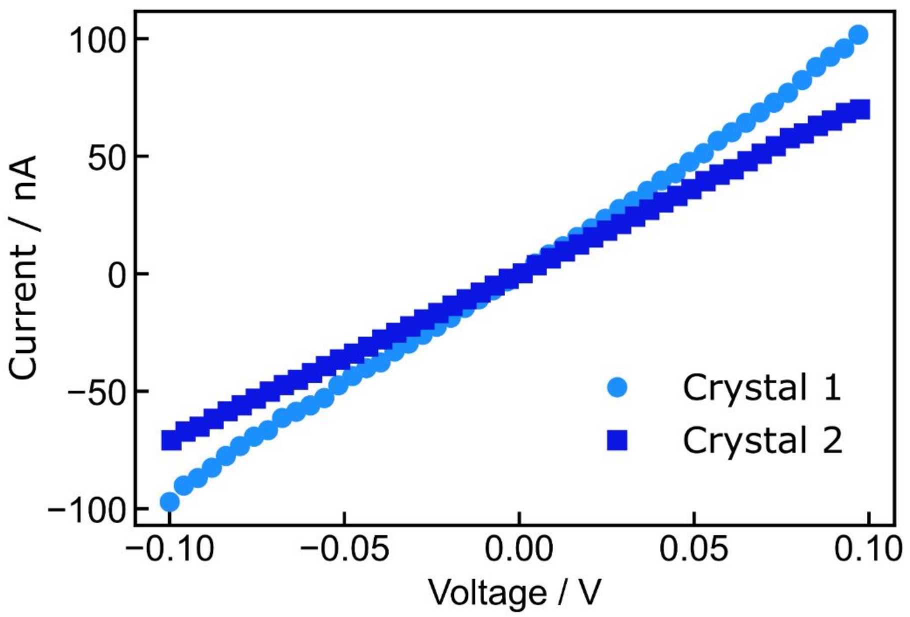

Fig. S10: *I*-*U* curves of two individual $Nb_{11}O_6I_{24}$-crystals at 300 K without illumination, showing Ohmic behavior.
